# Mortality following Stroke, the Weekend Effect and Related Factors: Record Linkage Study

**DOI:** 10.1371/journal.pone.0131836

**Published:** 2015-06-29

**Authors:** Stephen E. Roberts, Kymberley Thorne, Ashley Akbari, David G. Samuel, John G. Williams

**Affiliations:** 1 College of Medicine, Swansea University, Swansea, United Kingdom; 2 West Wales General Hospital, Carmarthen, United Kingdom; Örebro University, SWEDEN

## Abstract

**Background:**

Increased mortality following hospitalisation for stroke has been reported from many but not all studies that have investigated a ‘weekend effect’ for stroke. However, it is not known whether the weekend effect is affected by factors including hospital size, season and patient distance from hospital.

**Objective:**

To assess changes over time in mortality following hospitalisation for stroke and how any increased mortality for admissions on weekends is related to factors including the size of the hospital, seasonal factors and distance from hospital.

**Methods:**

A population study using person linked inpatient, mortality and primary care data for stroke from 2004 to 2012. The outcome measures were, firstly, mortality at seven days and secondly, mortality at 30 days and one year.

**Results:**

Overall mortality for 37 888 people hospitalised following stroke was 11.6% at seven days, 21.4% at 30 days and 37.7% at one year. Mortality at seven and 30 days fell significantly by 1.7% and 3.1% per annum respectively from 2004 to 2012. When compared with week days, mortality at seven days was increased significantly by 19% for admissions on weekends, although the admission rate was 21% lower on weekends. Although not significant, there were indications of increased mortality at seven days for weekend admissions during winter months (31%), in community (81%) rather than large hospitals (8%) and for patients resident furthest from hospital (32% for distances of >20 kilometres). The weekend effect was significantly increased (by 39%) for strokes of ‘unspecified’ subtype.

**Conclusions:**

Mortality following stroke has fallen over time. Mortality was increased for admissions at weekends, when compared with normal week days, but may be influenced by a higher stroke severity threshold for admission on weekends. Other than for unspecified strokes, we found no significant variation in the weekend effect for hospital size, season and distance from hospital.

## Introduction

Many studies that have investigated mortality following stroke according to the week day of admission have reported increased mortality on weekends [1–10], although other studies have found no increased mortality [11–21]. Possible reasons for the so called ‘weekend effect’ on mortality include a higher stroke severity threshold for admission on weekends, with milder strokes less often admitted [9,10], delays in investigations, treatment or access to specialist stroke services [6,9,22], reduced medical cover or staffing [4,21] and differences in the levels of patient co-morbidities among patients admitted on weekends and weekdays [3,7,10].

There have been major improvements over time in stroke services in the UK, assisted by findings from successive rounds of major national audits of stroke care run by the Royal College of Physicians since 1998 [23], and 2010 [9,24], and also following national clinical guidelines for stroke published in 2012 [25]. However, it is not clear as to how any reductions in stroke mortality may alter any 'weekend effect' on stroke mortality. Little is also known about the whether any weekend effect may be influenced by environental factors including, firstly, the size of the admitting hospital with possible variation in senior consultant and staffing cover at weekends [26]. Secondly, seasonal factors such as winter months which can impact on inpatient resources through seasonal increases in admissions for illnesses including chronic obstructive pulmonary disease,[27,28] respiratory infections [4,28,29] and norovirus [30]. Thirdly, the distance travelled to hospital by patients which could lead to delays in clinical investigations or treatment [31]. This study was designed to address this lack of evidence on the weekend effect.

The main objectives of this study were firstly to establish any changes over time in mortality following stroke. Secondly, whether there is increased mortality for admissions on weekends and public holidays when compared with week days and whether any 'weekend effect' on mortality has changed over time. Thirdly, whether any weekend effect may be related to factors including the size of the hospital, seasonal factors and distance from hospital.

## Methods

### Study design and setting

To investigate mortality following stroke, we used systematic record linkage of national inpatient, mortality and primary care data. The hospital inpatient data (Patient Episode Database for Wales) includes abstracts of inpatient admissions to all public National Health Service (NHS) hospitals across Wales, UK (population 3.07 million in 2012). The inpatient data were systematically linked together to enable subsequent admissions for the same people to be determined. They were also linked to national mortality data from the Office for National Statistics (ONS) and the NHS Welsh Administrative Register to identify all deaths that occurred following discharge from hospital as well as deaths that occurred as inpatients. Additionally, to identify patient co-morbidities from primary care as well as inpatient care, the inpatient and mortality data were also record linked to data available from 35% of all general practices across Wales during the entire study period.

The record linked inpatient, mortality and primary care data in this study are held as part of the Secure Anonymised Information Linkage (SAIL) databank and CIPHER (Centre for Improvement in Population Health through E-records Research), a component of the MRC Farr Institute of Health Informatics Research. As described and validated previously, the record linkage, principal inpatient diagnoses and mortality ascertainment have been validated previously and have been shown to be respectively >99.8%, >90% and >98% accurate [32–34].

### Ethics statement

Ethical approval and patient consent for the study was not required (as advised by the National Research Ethics Service (NRES) as the study data are fully anonymised. Study approval was obtained instead from the Information Governance Review Panel (IGRP) which is represented by NRES, the British Medical Association Ethics Advisor, the Caldicott Guardian and NHS Wales Informatics Service. The study data are publicly available to other researchers.

### Study inclusion and exclusion criteria

The study cohort included inpatient admissions where stroke was recorded as the principal diagnosis on the discharge record, from January 1^st^ 2004 to December 31^st^ 2011, with follow up to December 31^st^ 2012. The study included each person's first admission for stroke during the study period, followed them up for 365 days and included subsequent admissions for stroke provided they occurred at least 365 days after the preceding admission. Our study cohort should not be affected substantially by attrition over the one-year follow-up, as annual population emigration from Wales varied between 2.1% and 2.7% during the study period [35], and is lower among older people who are most often affected by stroke [35]. The International Classification of Diseases tenth revision (ICD-10) codes used for stroke were I61-I64.

### Week day of admission for stroke

Mortality according to the day of admission was assessed, firstly, by comparing mortality for admissions on weekends (Saturday 00:00 hours to Sunday 23:59 hrs) and public holidays (eight days each year) with normal weekdays (Monday 00:00 hours to Friday 23:59 hours) and, secondly, across each day of the week.

### Exposure measures

Study exposure measures or variables that may affect a possible weekend effect on mortality include the size of the admitting hospital [26], seasonal factors [4,27–30] and distance from hospital [31]. Hospital size was based on the total number of beds and defined as 'small' (100–399 beds), 'medium' (400–599 beds), 'large' including teaching hospitals (600+ beds) and other small ‘community’ or cottage hospitals (<100 beds).

Seasonal factors were assessed by comparing mortality during winter (December to February), spring (March to May), summer (June to August) and autumn (September to November). Distances from patients' residences to main hospitals (small to large) were measured in four categories; <5 kilometers (km), 5–9.99 km, 10–19.99 km and 20+ km and also as median distances.

Social deprivation was measured using the widely used Welsh Index of Multiple Deprivation (WIMD) 2008 version [36], which is similar to the English Indices of Multiple Deprivation (IMD) [37]. It includes seven domains of deprivation, ‘income’, ‘employment’, ‘education’, ‘health’, ‘access to services’, ‘housing’, ‘physical environment’ and ‘community safety’. The total WIMD deprivation scores for 1896 geographical Lower Super Output Areas (LSOAs) (average LSOA population = 1580), were ranked and categorised into quintiles (I = least deprived and V = most deprived quintile) [36]. Stroke subtype was based on the principal diagnosis at discharge using the following ICD-10 codes; cerebral infarction (I63), intracerebral haemorrhage (I61), other non-traumatic intracranial haemorrhage (I62) and stroke, unspecified (I64).

Patient mortality was adjusted for 11 major patient co-morbidities, based on diagnoses recorded in any diagnostic position during the patient’s current inpatient admission or from admissions or primary care consultations during the previous five years. The 11 patient co-morbidities were ischaemic heart disease, other cardiovascular diseases, cerebrovascular disease, other circulatory diseases, malignancies, liver disease, chronic obstructive pulmonary disease, asthma, diabetes, renal failure and dementia.

### Outcome measures and statistical analysis

The main study outcome measure was mortality at seven days following admission. Secondary outcome measures were mortality at 30 days and one year. Mortality rates were calculated using the numbers of admissions for stroke as the denominators and the numbers of deaths (from all causes) as the numerators.

Multivariate logistic regression was used to assess changes over time in mortality following stroke and to determine the influence of each of the study exposure factors on any 'weekend effect'. Analyses were adjusted for patient age group, sex and the 11 major patient co-morbidities. To eliminate any possible biases in the determination of patient co-morbidities from inpatient admissions and primary care consultations, we also adjusted for patients with no previous inpatient admissions or primary care consultations. Interaction effects on mortality between weekend admissions and the study factors were also investigated through logistic regression. There were no missing data for any of the study variables, except social deprivation (available for all 36 974 people who were resident in Wales; 97.6% of all cases) and distance from hospital (available for all 35 627 or 94.0% of people who were both resident in Wales and admitted to a main hospital). The 'missing cases' were excluded from the logistic regression modeling for, respectively, social deprivation and distance to hospital. Other statistical methods include the t-test, the Kruskal-Wallis test and the chi square test to compare the baseline characteristics of patients who were admitted for stroke on weekends, public holidays and week days. Significance was measured, firstly, at the conventional 5% level and, secondly, after applying a Bonferroni adjustment for multiple testing.

## Results

Of 37 888 people hospitalised for stroke, a majority (53%) were female. The mean age of the patients was 75.7 years (SD = 12.7; range = 18–109 years). Table 1 provides baseline summary characteristics of the patients who were admitted on normal week days, on weekends and public holidays. These three patient groups were similar in terms of their demographics, social deprivation, season and time period of admission, length of inpatient stay and patient co-morbidities (Table 1).

**Table 1:** Baseline characteristics of patients hospitalised for stroke on weekdays, weekends and public holidays, 2004 to 2012.

	Week days		Weekends		Public holidays		p-value
	No. of cases‡	(%)‡	No. of cases‡	(%)‡	No. of cases‡	(%)‡	
**Patient age** (mean + SD)	75.8	(+ 12.6)	75.6	+ 12.8	75.1	+ 12.9	0.184
**Patient Sex**							
Men	13 271	(47.2%)	4309	(47.8%)	356	(47.7%)	0.636
**Hospital size**							
Small (100–399 beds)	4683	(16.7%)	1687	(18.7%)	138	(18.5%)	<0.001
Medium (400–599)	12 756	(45.4%)	4247	(47.1%)	354	(47.7%)	
Large (600+)	8434	(30.0%)	2688	(29.8%)	218	(29.2%)	
Community (<100)	2246	(8.0%)	400	(4.4%)	37	(5.0%)	
**Time period of admission**							
2004–2005	7182	(25.5%)	2348	(26.0%)	168	(22.5%)	0.196
2006–2008	10 480	(37.3%)	3395	(37.6%)	290	(38.8%)	
2009–2011	10 447	(37.2%)	3279	(36.3%)	289	(38.7%)	
**Season of admission**							
Winter	6891	(24.5%)	2217	(24.6%)			0.404
Spring	6997	(24.9%)	2319	(25.7%)			
Summer	7175	(25.5%)	2259	(25.0%)			
Autumn	7056	(25.1%)	2227	(24.7%)			
**Patient distance from hospital**							
Median km (IQ range)	8	(4–15)	8	(4–15)	8	(4–15)	0.010
**Patient social deprivation**							
I	4829	(17.6%)	1456	(16.6%)	105	(14.5%)	0.116
II	5074	(18.5%)	1588	(18.1%)	141	(19.4%)	
III	5948	(21.6%)	1974	(22.5%)	156	(21.5%)	
IV	5816	(21.2%)	1836	(20.9%)	165	(22.7%)	
V	5814	(21.2%)	1913	(21.8%)	159	(21.9%)	
**Hospitals with formal stroke unit**							
Yes	1498	(5.3%)	405	(4.5%)	44	(5.9%)	0.005
No	26 621	(94.7%)	8617	(95.5%)	703	(94.1%)	
**Stroke subtype**							
Cerebral infarction	16 955	(10.3%)	5469	(11.6%)	459	(12.6%)	0.001
Intracerebral haemorrhage	2899	(4.2%)	1048	(3.8%)	94	(4.3%)	
Other non-traumatic intracranial haemorrhage	1170	(60.3%)	346	(60.6%)	32	(61.4%)	
Unspecified stroke	7095	(25.2%)	2159	(23.9%)	162	(21.7%)	
**Specialty of first consultant**							
Stroke or geriatric medicine	4288	(15.2%)	1185	(13.1%)	99	(13.3%)	<0.001
General medicine	16 548	(58.8%)	5344	(59.2%)	463	(62.0%)	
Neurosurgery	174	(0.6%)	55	(0.6%)	2	(0.3%)	
General surgery	141	(0.5%)	47	(0.5%)	3	(0.4%)	
Other	7109	(25.3%)	2391	(26.5%)	183	(24.5%)	
**Length of inpatient stay**							
Median days (IQ range)	10	(4–24)	10	(4–24)	11	(4–24)	0.496
**Patient co-morbidities**							
Malignancies	3759	(13.4%)	1262	(14.0%)	96	(12.9%)	0.283
Ischaemic heart disease	8971	(31.9%)	2896	(32.1%)	225	(30.1%)	0.535
COPD	3595	(12.8%)	1208	(13.4%)	98	(13.1%)	0.326
Dementia	3304	(11.8%)	1054	(11.7%)	91	(12.2%)	0.918
Liver disease	513	(1.8%)	174	(1.9%)	15	(2.0%)	0.775
Renal failure	2814	(10.0%)	882	(9.8%)	71	(9.5%)	0.751
Diabetes	5785	(20.6%)	1849	(20.5%)	150	(20.1%)	0.939
Hypertension	18 640	(66.3%)	5870	(65.1%)	489	(65.5%)	0.097

Notes

‡ Numbers of cases and % of cases are reported unless otherwise denoted in the left most column.

Hospital transfers were less common (p<0.001) on weekends (247; 2.7% of all admission on weekends) and public holidays (26; 3.4%) than on week days (1640; 5.8%).

On weekends, compared with normal weekdays, stroke patients were more often admitted to small and medium size hospitals rather than large hospitals (p<0.001), were less frequently managed first by stroke or geriatric medicine specialties (p<0.001). The overall admission rate for stroke was higher on normal week days (111 per 100 000 population; 95% CI = 110–113) than on weekends (88; 86–90) and public holidays (95; 88–102).

### Mortality following stroke

Overall mortality following stroke during the nine year study period was 11.6% at seven days, 21.4% at 30 days and 37.7% at one year. Mortality at seven days fell by 14% from 2004 to 2012 (mean annual reduction = 1.7% (95% CI = 0.3%-3.0%) and mortality at 30 days fell by 25% (mean annual reduction = 3.1%; 2.0%-4.1%).

### Mortality for admissions on weekends

For admissions on weekends, compared with admissions on normal weekdays, mortality adjusted for patient demographics and co-morbidities was increased by 19% (95% CI = 10%-29%) at seven days and by 15% (8%-22%) at 30 days (Table 2). Adjusting also for the size of the hospital and the specialty of the first consultant reduced the weekend effect a little, but not significantly, at seven days (increased by 15%; 7%-23%) and at 30 days (12%; 6%-19%).

**Table 2:** Mortality at 7 and 30 days following hospital admission for stroke according to patient demographics and week day of admission, 2004 to 2012.

		Mortality at 7 days			Mortality at 30 days		
	No. of admissions	Mortality rate	Adjusted mortality odds ratio†	(95% CI)	Mortality rate	Adjusted mortality odds ratio†	(95% CI)
**All cases**	37 888	11.6% 12%			21.4%		
**Patient age group**							
<35	234	8.1%			10.3%		
35–44	591	7.4%			10.3%		
45–54	1775	8.0%			10.4%		
55–64	4179	7.9%			11.2%		
65–74	7989	9.5%			14.9%		
75–84	13 171	11.7%			21.6%		
85+	9949	15.7%			33.4%		
**Patient sex**							
Male	17 936	9.8%	Ref	**-**	17.6%	Ref	**-**
Female	19 952	13.1%	1.25	(1.16, 1.33)*	24.7%	1.27	(1.20, 1.34)*
**Day of admission**							
Weekdays	28 119	11.1%	Ref	-	20.7%	Ref	-
Weekends	9022	12.9%	1.19	(1.10, 1.29)* 291.28)*	22.9%	1.15	(1.08, 1.22)*
Public holidays	747	13.3%	1.24	(1.00, 1.54)	25.3%	1.34	(1.12, 1.59)*
Monday	6072	11.3%	Ref	-	20.5%	Ref	-
Tuesday	5951	11.2%	0.99	(0.88, 1.11)	21.4%	1.05	(0.96, 1.15)
Wednesday	5602	11.0%	0.97	(0.86, 1.09)	20.3%	0.98	(0.89, 1.07)
Thursday	5513	11.5%	1.01	(0.90, 1.14)	21.2%	1.01	(0.93, 1.11)
Friday	5681	10.9%	0.96	(0.86, 1.08)	20.7%	1.01	(0.92, 1.10)
Saturday	4591	12.3%	1.10	(0.97, 1.24)	22.9%	1.14	(1.04, 1.26)
Sunday	4478	13.5%	1.24	(1.10, 1.40)*	23.1%	1.18	(1.07, 1.29)*

Notes

Ref = Reference category

† The mortality odds ratio for patient sex is adjusted for patient age group and 11 patient co-morbidities, the mortality odds ratios for all other factors are adjusted for patient age group, sex and the patient co-morbidities. The 11 patient co-morbidities with ICD-10 codes are ischaemic heart disease (120-I25); other cardiovascular diseases (I00-I15, I26-I52); cerebrovascular disease (I60-I69); other circulatory diseases (I70-I99); malignancies (C00-C97); liver disease (K70-K77); chronic obstructive pulmonary disease (J40-J44); asthma (J45, J46); diabetes (E10-E14); renal failure (N17-N19) and dementia (F00-F03, F05.1, G30).

* Denotes significance after applying a Bonferroni adjustment

For admissions on public holidays, compared with normal week days, adjusted mortality was increased by 24% (0%-54%) at seven days and by 34% (12%-59%) at 30 days. Mortality was highest for admissions on Sundays, followed by admissions on Saturdays (Table 2).

### Factors that may increase mortality for weekend admissions

When assessing how the study factors may influence or modify the weekend effect on mortality, formal interaction testing showed that the only factor with significant variation in mortality was stroke subtype (p = 0.009 for mortality at seven days and p = 0.021 for mortality at 30 days). Compared with cerebral infarction admissions on weekends, mortality for unspecified stroke admissions on weekends was increased significantly by 32% at seven days and by 21% at 30 days.

Of the other study factors, the increased mortality at seven days for admissions on weekends was apparent for both male and female patients (23% increase for men and 18% for women; Table 3). The weekend effect was evident for all patient age groups of 65+ years and fell slightly but non-significantly to 14% during the most recent study years from 2009 to 2011 (Table 3).

**Table 3:** Mortality at 7 and 30 days for weekend admissions compared with week days, according to study factors, 2004 to 2012.

		Mortality at 7 days		Mortality at 30 days	
	No. of admissions	Adjusted mortality odds ratio (weekends: week days)†	(95% CI)	Adjusted mortality odds ratio (weekends: week days)†	(95% CI)
**All cases**	37 888	1.19	(1.10, 1.28)*	1.16	(1.10,1.24)*
**Patient age group**					
<65	6779	1.16	(0.95, 1.43)	1.10	(0.92, 1.31)
65–74	7989	1.25	(1.05, 1.48)	1.12	(1.02, 1.24)
75–84	13 171	1.17	(1.04, 1.31)	1.27	(1.10, 1.46) 1.28)*
85+	9949	1.19	(1.05, 1.35)	1.15	(1.04, 1.27)
**Patient sex**					
Male	17 936	1.23	(1.10, 1.37)	1.13	(1.03, 1.23)
Female	19 952	1.18	(1.08, 1.31)*	1.19	(1.10, 1.29)*
**Hospital size**					
Small (100–399 beds)	6508	1.15	(0.97, 1.37)	1.02	(0.88, 1.17)
Medium (400–599)	17 357	1.18	(1.06, 1.31)	1.21	(1.11, 1.32)*
Large (600+)	11 340	1.08	(0.95, 1.23)	1.09	(0.98, 1.21)
Community (<100)	2683	1.81	(1.12, 2.91)	1.31	(0.96, 1.79)
**Time period of admission**					
2004–2005	9696	1.17	(1.01, 1.35)	1.08	(0.96, 1.22)
2006–2008	14 165	1.19	(1.07, 1.33)	1.25	(1.14, 1.37)*
2009–2011	14 025	1.14	(1.00, 1.29)	1.13	(1.02, 1.25)
**Season of admission**					
Winter	9391	1.31	(1.14, 1.50)*	1.22	(1.09, 1.37)*
Spring	9684	1.11	(0.96, 1.28)	1.05	(0.94, 1.18)
Summer	9530	1.17	(1.01, 1.36)	1.14	(1.01, 1.28)
Autumn	9283	1.18	(1.01, 1.36)	1.21	(1.07, 1.36)
**Patient distance from hospital**					
<5 km	10 333	1.13	(0.98, 1.30)	1.08	(0.96, 1.21)
5 km–9.9 km	9587	1.13	(0.97, 1.31)	1.17	(1.04, 1.32)
10 km–19.9 km	10 514	1.15	(1.01, 1.32)	1.18	(1.06, 1.32)
20+ km	5193	1.32	(1.11, 1.57)	1.08	(0.93, 1.25)
**Patient social deprivation**					
I	6390	1.18	(0.98, 1.42)	1.18	(1.02, 1.37)
II	6803	1.45	(1.23, 1.73)*	1.30	(1.13, 1.49)*
III	8078	1.20	(1.03, 1.40)	1.14	(1.00, 1.29)
IV	7817	1.16	(0.99, 1.37)	1.11	(0.97, 1.27)
V	7886	1.03	(0.88, 1.21)	1.09	(0.96, 1.25)
**Hospitals with formal stroke unit**					
Yes	1947	1.23	(0.81, 1.88)	1.18	(0.87, 1.62)
No	35 941	1.20	(1.11, 1.29)*	1.16	(1.09, 1.23)*
**Stroke subtype**					
Cerebral infarction	22 883	1.06	(0.90, 1.25)	1.04	(0.89, 1.21)
Intracerebral haemorrhage	4041	1.07	(0.94, 1.21)	1.08	(0.99, 1.18)
Other nontraumatic intracranial haemorrhage	1548	1.23	(0.89, 1.68)	1.27	(0.96, 1.67)
Unspecified stroke	9416	1.39	(1.22, 1.58)*	1.31	(1.17, 1.46)*

Notes

Ref = Reference category

† The mortality odds ratios are adjusted for patient age group, sex and the patient co-morbidities

* Denotes significance after applying a Bonferroni adjustment

Mortality was lower in the hospital that provided a formal stroke unit throughout the study period (6.8%; 95% CI = 5.7%-8.0%) than in all other main hospitals: mortality was 9.5% (8.1%-11.1%) in the hospital with the next lowest mortality. There was evidence of higher mortality for weekend admissions for this hospital (23% increase) and for all others (20%) with no significant variation in the weekend effect.

The weekend effect on mortality was highest in community hospitals (81% increase and lowest in large hospitals (8% increase) although with relatively few admissions to community hospitals there was no significant variation according to hospital size. Although not significant, the weekend effect was also slightly stronger during the winter months (31%) compared with spring (11%), summer (17%) and autumn (18%), for patients resident furthest from hospital (≥20 km, 32% increase) and for patients who were moderately affluent, social deprivation quintile II (45%). There were also highly significant associations (p<0.001) between social deprivation, patient distance from hospital and the size of the hospital.

After applying a Bonferroni adjustment for multiple testing, the weekend effect at seven days was still significant for the following: all stroke patients, patients aged 65–84 years, male and female patients, admissions from 2006 to 2008, winter admissions, social deprivation quintile II, hospitals with no formal stroke unit and unspecified stroke subtype (Table 3).

## Discussion

The study shows that mortality following stroke has fallen strongly over time. Mortality was increased for admissions at weekends, when compared with normal week days, but may be influenced by a higher stroke severity threshold for admission on weekends, especially as the admission rate was substantially lower on weekends. This study was designed to establish how any increased mortality for weekend admissions may be affected by several factors. Although there were some indications that the weekend effect on mortality at seven days was higher during the winter months, in community hospitals rather than in large hospitals and for stroke patients who were resident furthest from hospital, these differences were not significant.

The increased mortality for weekend admissions of 19% at seven days is very similar to a corresponding figure of 18% reported nationally across England during the 12 month period from April 2009 to March 2010 [6] and our increased weekend mortality of 16% at 30 days is also similar to 14% reported from the 130 hospitals across England included in a recent national audit from 2010 to 2012 [9].

Although there was no significant variation in the weekend effect according to the season of the year, mortality at seven days for weekend, compared with weekday admissions was 31% higher during winter months. During the winter, emergency hospital admissions are often increased through seasonal respiratory and gastrointestinal infections including influenza and norovirus, as well as chronic conditions such as COPD, which lead to increased pressures on inpatient resources. It is possible that stroke care may be compromised in hospitals without dedicated stroke units, although we found no significant variation in the weekend effect.

The weekend effect was highest in community hospitals (80% increase)—although because of relatively few admissions to these hospitals, this was not statistically significant—compared with large hospitals (10%). Community and small hospitals can be most affected by a lack of access to investigation, treatment and senior cover at weekends, while larger hospitals are often better able to address these shortcomings. Patients in community hospitals were significantly older (mean age 79.0 years) than in small (76.0 years), medium (75.4) and large hospitals (75.2), while a previous UK audit reported that the quality of acute stroke care is worse among older patients [22].

Although many of the study hospitals provide informal stroke units that involve reserved beds, only one provided a formal, dedicated stroke unit throughout the study period. Mortality was significantly lower in this hospital (7.0% at seven days) than in all other hospitals. However, the small number of admissions was insufficient to establish whether formal stroke units are effective in overcoming the weekend effect, as reported in other studies [17–19]. It is also possible that the findings in our population may differ from other national populations which have higher levels of admissions to formal stroke units.

We found that the weekend effect was substantially increased for strokes that were recorded as unspecified subtype. This suggests that the weekend effect on mortality is worsened for strokes on weekends that are less well investigated or recorded—and may reflect lower levels of service provision and staffing on weekends.

There were indications, although not statistically significant, that the weekend effect on stroke mortality at seven days may be higher for patients who lived furthest from hospital, with 32% increased mortality for distances of more than 20 km, and for social deprivation quintile II (45% increased). Longer distances to hospital can lead to delays in the initiation of investigations and treatment.[31] The indications of an increased weekend effect for social deprivation quintile II is less intuitive, but may be linked to strong associations between social deprivation and both distance from a main hospital and the size of the admitting hospital. Patients in quintile II lived furthest from a main hospital and were most often admitted to a community hospital, which were also both linked with some indications of increased mortality for admissions at weekends. This finding may be particular therefore to the study population and the geographical dispersion of affluent and deprived communities in relation to the location of hospitals.

A strength of this study is that it covers more than 37 000 admissions for stroke in a defined geographical population. It is based on systematic, validated record linkage of inpatient and death certificate data to identify all deaths that occurred following discharge from hospital as well as those that occurred in hospital. It also incorporated validated record linkage of national primary care data to enable more complete ascertainment of patient co-morbidities than in other large studies of stroke mortality that have been based on co-morbidities recorded from inpatient records alone.

Study limitations are, firstly that as with other studies of stroke mortality, based on administrative health data [1–6,8,10,11,13,14,17] and which provide most of the evidence on the weekend effect, our information sources lack detailed information about stroke severity, disease history and treatment. Weekend admissions can include a higher proportion of more severe strokes with worse prognosis [9,23,24]. Although the weekend effect could not be explained by differences in case mix factors such as patient co-morbidities, demographics, social deprivation and length of inpatient stay, the admission rate was lower on weekends, suggesting that stroke severity may be greater on weekends. Other limitations, common to other large studies based on administrative health data [1–6,8,10,11,13,14,17] are that the study was restricted to public (NHS) hospitals, although the private hospital sector receives few admissions for stroke. Also, the measures of prognosis reported here are those from the index admission of stroke and we recognise that some of these may occur late in the natural history of the disease. The ascertainment of patient co-morbidities, although enhanced through primary care data, would be incomplete. Distances from hospital were measured from patients' residences rather than the locations of the strokes. As with other studies based on administrative inpatient data [1–6,8,10,11,13,14,17] our information sources included the day of admission but not the time of day the admission occurred, so that it was not possible to establish whether the weekend effect was linked to particular shifts. Although a conservative adjustment, significant findings would still be expected for multiple testing in a large population when using the Bonferroni adjustment.

The increased mortality for weekend and public holiday admissions in our study period amounts to an excess of 1,057 deaths at seven days and 1,941 at 30 days, when compared with mortality at the same levels as on normal weekdays. However, there were major reductions over time in stroke mortality, as stroke care has improved following successive national audits [9,23,24].

The study shows a strong reduction over time in mortality following hospitalisation for stroke, which follows the findings of national audits into stroke care. Mortality was increased for admissions at weekends, although a lower admission rate on weekends suggests that the weekend effect may well be influenced by variation in stroke severity. We found that the weekend effect on mortality was worse for strokes that were recorded as of 'unspecified' subtype. There were also indications, although not significant, that mortality for weekend admissions may be higher during the winter months when seasonal illnesses can impact most strongly on health care resources, for small community hospitals and for longer distances to hospital. Further studies are required in other healthcare settings in order to establish whether the weekend effect on stroke mortality is worsened during the winter, in community hospitals and for people resident furthest from hospital.
